# “Walking While Black”: Impacts of Inequities in Nature Access and Belongingness

**DOI:** 10.1007/s40572-026-00554-6

**Published:** 2026-08-06

**Authors:** Jennifer D. Roberts, Sasha Hali Tolliver-Chavez, Roger Isom

**Affiliations:** 1 Wekesa Earth Center, University of Maryland, College Park, MD 20742-2611 USA; 2 Physical Cultural Studies Research Group, Department of Kinesiology, University of Maryland, SPH Building #255, 4200 Valley Drive, College Park, MD 20742-2611 USA

**Keywords:** “Walking While Black”, Nature access, Nature belongingness, Racialized public space, Health inequity, Health disparity

## Abstract

**Review Purpose:**

As a framework for understanding inequities in nature access and belongingness among Black Americans, this review examines the historical and public health significance of “Walking While Black.” By tracing the colloquial evolution of "Walking While Black," contemporary experiences within a broader history of racialized surveillance and exclusion are positioned.

**Recent Findings:**

Recent research indicates that inequities in nature access extend beyond the physical availability of parks and green spaces to include safety, inclusion, and belongingness. Historical and contemporary forms of racial exclusion have limited outdoor engagement for many Black Americans. These inequities are associated with reduced physical activity, chronic stress, anxiety, hypervigilance, environmental injustice, and diminished opportunities for social connection and community engagement.

**Review Summary:**

“Walking While Black” reflects the enduring racialization of mobility and presence in public and natural spaces. The resulting barriers to nature access and belongingness contribute to physical, mental, emotional, and social health disparities among Black Americans. Achieving environmental and health equity requires both expanding access to green spaces and creating environments where Black individuals can safely and freely exist without fear, suspicion, or exclusion.

## Introduction: “Walking While Black”

Inarguably, walking is one of the most accessible forms of physical activity and a cornerstone of public health. However, as suggested by the idiom, “Walking While Black,” the ability to walk safely and freely is an unrecognized active living privilege. For many Black Americans^1^ and non-White presenting individuals, walking in outdoor environments is and has been shaped by historical injustices, structural inequities, and racialized experiences. This review journeys back in time, through the tectonic derivation of the colloquialism and examines the impacts of walking, moving, or just existing as a Black American in nature and open spaces. From the fields of southern cotton plantations filled with African slaves, to “Giving Whites The Wall,” an enforcement of racial hierarchy, subjugation, and White supremacy, and on to the 2020 Christian Cooper incident, an example of weaponized Whiteness in New York City’s Central Park, this historical account will paint an intimate understanding of the malfeasance, fear, intimidation, and underlying maladies of “Walking While Black.”

## “Walking While Black”: Evolution of Expression

**Fig. 1:**
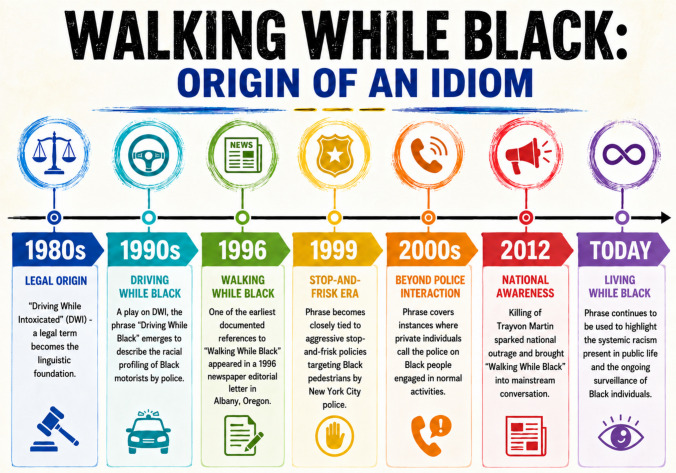
“Walking While Black”: Origin of an Idiom

“Walking While Black,” a modern colloquial phrase in American English, evolved from the more commonly known expression “Driving While Black.” During the 1990 s, as evidence mounted that Black motorists in the United States were disproportionately stopped and searched by police compared to White motorists, the phenomenon of “Driving While Black” gained national attention [1–3]. Areas throughout the country, particularly urban environments were plagued by the racial profiling of state troopers and city police. The idiom was a play on the phrase “Driving While Intoxicated,” and it emphasized how Blackness alone could become grounds for suspicion. Newspapers routinely reported examples of “Driving While Black” while authorities continuously denied the profiling and justified their actions. When a 26-year-old Black man said he was stopped on 26 occasions from the age of 16 years by the Indianapolis police, the Deputy Police Chief replied, “We stop everybody. If it don’t look right, it don’t look right” [4]. By the mid to late 1990s, the phrase expanded beyond automobiles to include ordinary pedestrian activity. One of the earliest documented references to “Walking While Black” appeared in a 1996 newspaper editorial that described the author’s victimization of this profiling [5]. Then, three years later, the late Johnnie Cochran^2^ was quoted as saying “it’s walking while Black, jogging while Black…indeed, it’s living while Black” [6]. That same year, “Walking While Black” became an issue of particular notice in New York City when Mayor Rudolph Giuliani and Police Commissioner Howar Safir were accused of violating the civil rights of citizens through their stop-and-frisk practices [7]. Critics argued that Black pedestrians were being targeted simply for existing in public space. Over the next decade or so, the expression continued to suffuse the concrete jungles of cities and urban environments, but more recently it has been unfurled to parks, greenways and other natural settings. “Walking While Black” became more than a phrase about police stops; it evolved into a broader critique of how public spaces are racialized and how walking for many Black Americans has required constant vigilance and self-monitoring to avoid being perceived as a criminal or dangerous (Fig. 1).

## “Walking While Black”: Historical Markers

### Slave Patrols and Paddy Rollers

On March 2, 1807, President Thomas Jefferson signed into law the 'Act Prohibiting the Importation of Slaves.' Taking effect on January 1, 1808, this legislation ended the legal entry of enslaved people to the United States, but it did not abolish slavery itself. By this point, approximately 280,000 Black bodies had been pilfered from the continent of Africa and brought to the country [8]. The practical impact of the Act was inconsequential because the domestic slave trade within the United States was already sustained by the natural growth of the “inventory” or enslaved population. Now, involvement in international slave trade was outlawed, but internal trade flourished with the tripling of enslaved persons over the next five decades [9]. In 1860 there were nearly four million enslaved bodies of which two million were living on cotton plantations in southern American states [9]. Notably, the American Confederacy^3^ (South Carolina, North Carolina, Mississippi, Florida, Alabama, Georgia, Louisiana, Arkansas, Virginia, Tennessee, Texas) held the backbone of the plantation economy, which fueled the country’s growth and prosperity through chattel slavery. Other states, such as Maryland and areas throughout the North, that were not part of the Confederacy capitalized on this system [10]. Many northern businesses, such as textile factories, shipping businesses, banking firms, and insurance companies, were enriched by the southern enslaved labor force [11, 12]. Collectively, the ownership and labor of enslaved “property” were investments that generated immense wealth, reinforced systems of racial hierarchy, and fueled the economic foundation of America. However, another equally important facet of this entire enterprise was becoming firmly established; the policing of Black bodies through legal and enforcement systems.

**Fig. 2:**
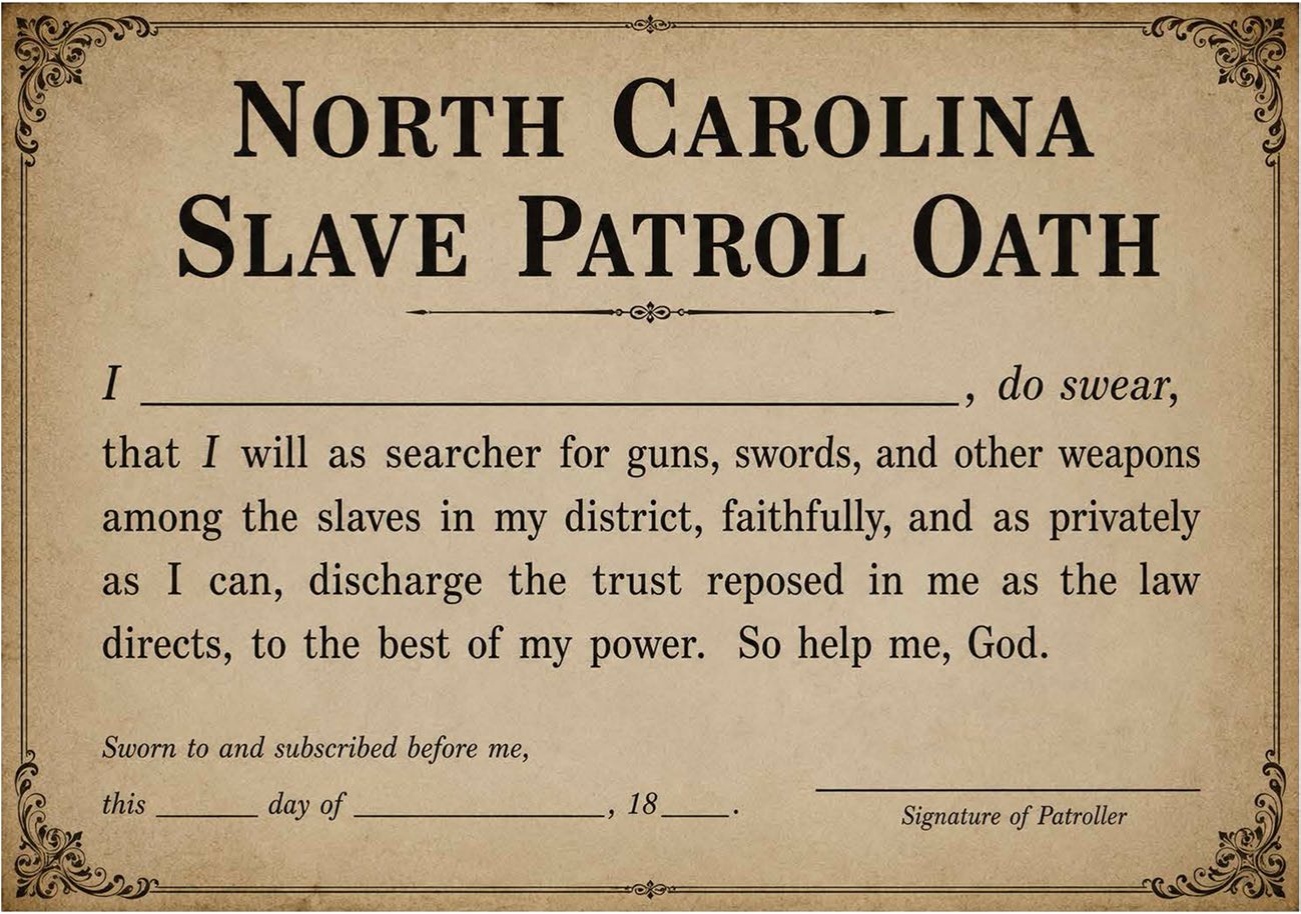
Slave Patrols

Antebellum groups of armed White men known as slave patrols or “paddy rollers” were state-sanctioned organizations instituted to pursue, apprehend, and return enslaved escapees to their enslavers (Fig. 2). Established in the early 1700 s, first in South Carolina, slave patrols monitored and disciplined, deterred insurrections through organized terror and enforced strict “slave codes” that controlled the movements and actions of the enslaved [13]. These vigilante posse-type groups of approximately ten men and sometimes women were paid to patrol specific areas [14]. Supported by the legal framework of fugitive slave laws, patrols could enter, without warrant or permission, the homes of anyone, including White residents, who were suspected of harboring fugitives. These laws, particularly the Fugitive Slave Act of 1850, transformed the capture and return of escaped enslaved people into a federally protected legal obligation and compelled citizens and local authorities throughout the country to assist in the recovery of human property. By denying alleged fugitives basic legal protections such as jury trials or the right to testify on their own behalf, these laws expanded the power of patrols and enslavers while embedding slavery into the fabric of the American legal system. Slave patrols served not only as instruments of control but also as symbols of racial hierarchy. Poor and working-class White men who did not own enslaved people were often recruited into patrols, which gave them an authority over Black individuals and reinforced a racial solidarity among the White community [15]. This “social wage,” or as referred to by W.E.B. DuBois “public and psychological wage,” demonstrates how slave patrols were more than financially compensated, but how they received intangible, non-monetary social status and privileges based on their racial classification [16]. With 7% of the total southern population “own[ing] nearly 3 million of the 3,953,696 slaves” in 1860 and “rul[ing] five million White people,” the wealthy elite White enslavers, often referred to as the antebellum planter class, were an exclusive group [16]. It is important to note that during the colonial era and the period of enslavement, “Whiteness” functioned primarily as a legal and political category that distinguished Anglo-European colonists from enslaved Africans and Indigenous peoples. Colonial governments gradually codified racial distinctions through laws that granted privileges to those classified as White, including access to citizenship, land ownership, legal protections, and freedom from enslavement. In this early context, Whiteness was largely associated with English and northwestern European ancestry and served as a mechanism for maintaining racial hierarchy and social control [17, 18]. Colonial elites deliberately cultivated a White identity among Europeans to create solidarity across class divisions while reinforcing the subordination of Africans and Indigenous peoples [19]. Over the 19th and early 20th centuries, however, the boundaries of Whiteness expanded. Many immigrant groups now widely regarded as White, including people of Irish, Italian, Jewish, and other southern and eastern European backgrounds, were initially viewed by Anglo-American Protestants as ethnically, culturally, and sometimes even racially inferior [20, 21]. These groups often faced discrimination, exclusion, and questions about their fitness for citizenship and social inclusion. Over time, access to citizenship, political alliances and changing demographic conditions facilitated their incorporation into the broader category of Whiteness. This historical evolution demonstrates that Whiteness has never been a fixed biological reality, but rather a socially constructed and legally reinforced status. Furthermore, the ability to present as White, often referred to as "passing," has historically provided access to privileges associated with Whiteness, including reduced exposure to discrimination, greater social acceptance, enhanced economic opportunities and institutional power [22, 23]. In 2012, George Zimmerman leveraged such power. Zimmerman, a multiracial man of White and Hispanic descent, assumed the role of neighborhood watch patrol in a gated Florida community and viewed the presence of Trayvon Martin, a Black 17-year-old pedestrian as suspicious. Zimmerman followed and confronted Martin before fatally shooting the unarmed teenager [24]. The incident illustrates how the social authority associated with Whiteness or perceived Whiteness can extend beyond formal institutions and shape who is viewed as belonging, who is considered threatening, and who feels empowered to police public space. This is not dissimilar to the working-class White men during enslavement who found a way to elevate their social status via slave patrolling. By the end of the 18th century, every slave state where slavery was legal had state-sanctioned slave patrols [25].

The movement of enslaved bodies were regulated by legislation forbidding them to leave the plantation or enslaver’s land without a written certificate or pass granting permission. Through a system of surveillance, violence, and legal control, these written certificates, imposed by patrols, set an early precedent of “Walking While Black.” Any Black person, enslaved or not, walking on roads, gathering in groups, traveling at night, or appearing “out of place” could be stopped, interrogated, beaten, detained, or returned to an enslaver. Slave patrols racialized surveillance to which Black bodies in motion were viewed as threats to the social order. They perpetuated a racial hierarchy by controlling where Black bodies could walk, when they could walk, and how freely they could exist in public spaces. Thus, the relationship between slave patrols and the idea of “Walking While Black” lies in the continuity of racialized control over Black mobility. Since enslavement, open or public spaces have been racially coded and the presence of Black bodies in these spaces have often been criminalized or surveilled whereby “authorities” assume that Black movement requires monitoring or justification. It has been argued that slave patrols became one of the earliest forms of organized policing in the American South and heavily influenced later law enforcement institutions [15]. The similarities between the practices of patrols and modern American policing or everyday citizen racial profiling cannot be denied. One commonality of note is the geography of race. For example, slave patrols enforced boundaries between plantations, towns, and White spaces. Similarly, current day acts of racism (e.g., “Return to the Land”)^4^ and racial profiling (e.g., “Hiking While Black”) has often been intensified when Black individuals move through spaces socially coded as White or affluent [26, 27]. As such, “Walking While Black” can be understood as a contemporary manifestation of a historical system rooted in slave patrols and racialized policing.

### “Giving Whites The Wall”

During the era of legalized segregation in the United States, spanning from the late 1870 s to the mid-1960s, racial domination was enforced not only through laws and terror, but also through countless everyday customs designed to subjugate Black Americans and reinforce White supremacy. One such practice was known as “Giving Whites The Wall,” an unwritten rule of racial etiquette that required Black individuals to yield the safer or entire portion of sidewalks to White pedestrians. In many Jim Crow southern communities and in parts of the North, Black pedestrians were expected to step off the sidewalk entirely or walk closest to the street whenever a White person approached. The phrase “Giving Whites The Wall” referred to the expectation that White pedestrians should occupy the inside portion of the sidewalk nearest to the walls of buildings and storefronts, while Black pedestrians moved aside or walked nearer to the curb [28]. This seemingly mundane practice reflected a much larger system of racial hierarchy that governed public space, bodily movement, and social interaction. “Giving Whites The Wall” was not merely about foot traffic. Rather, it symbolized the social order of White dominance and Black subordination that defined everyday life in the United States. The sidewalk became a powerful symbol of racialized space because it represented American citizenship and belonging. By forcing Black pedestrians to surrender physical space to White pedestrians, Jim Crow customs communicated that Black Americans had no equal claim to public or outdoor environments. The act of stepping into the street for White individuals was a daily ritual of submission and demonstration of inferiority that many Black Americans endured. In towns across the country, failure to comply could result in verbal abuse, physical assault, arrest, or lynching. The custom therefore functioned as a mechanism of social control enforced through fear and violence [28].

**Fig. 3:**
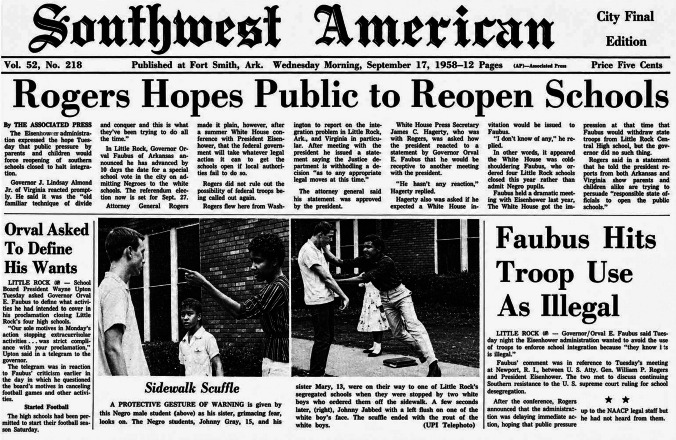
“Giving Whites the Wall”

“Giving Whites the Wall” also reinforced broader notions of racial purity and contamination embedded within segregationist ideology. White supremacists viewed Black proximity as threatening to the racial order. Consequently, maintaining physical distance became central to the rituals of Jim Crow society or the hierarchy of caste [29]. As delineated by Isabel Wilkerson, one of the eight pillars of maintaining a caste system is purity versus pollution whereby the superior group is inherently pure and the inferior group(s) are defiling or polluting. As a result, there had to be rigidly enforced rules regarding physical touch and proximity. Sidewalk etiquette reflected this obsession with racial hierarchy and spatial separation. The expectation that Black people move aside for White people communicated that White comfort and mobility were inherently more valuable. Importantly, these practices were enforced psychologically as well as physically. Jim Crow customs sought to normalize Black submission and White authority so completely that the racial hierarchy appeared natural and unquestionable. As White children expected dominance in these spaces, Black children learned these rules at an early age as strategies for survival [30]. Or in some cases, Black youth rebelled the expectation. In the late 1950 s, an adolescent boy in Little Rock, Arkansas named Johnny Gray punched a White student during a scuffle. Johnny and his sister, Mary, were en route to their segregated school when two White boys ordered them to get off the sidewalk (Fig. 3) [31]. The sidewalk became a site where racial terror and social conditioning intersected. The practice also revealed how White supremacy and legalized segregation extended beyond major institutions into the smallest details of ordinary everyday interactions and “Walking While Black.” The legacy of “Giving Whites the Wall” continues to resonate in modern discussions about race, policing, and public space. Contemporary expressions such as “Walking While Black” reflect ongoing concerns about the surveillance and regulation of Black movement in public environments. Although Jim Crow laws have been abolished and this expected racial etiquette is no longer the norm, it can be argued that racial profiling, disproportionate policing, and suspicion directed toward Black Americans in public spaces represent continuities with this earlier system of racial control. The historical practice of forcing Black pedestrians to surrender the sidewalk demonstrates how racial inequality was and still is embedded into the geography of everyday life and how movement itself became politicized under White supremacy.

### Weaponized White Privilege

In May 2020, amid a national reckoning over race and policing in the United States, Christian Cooper, while birdwatching in Central Park, fell victim to weaponized White privilege. The incident began in an area of Central Park known as the Ramble, where dogs are required to be leashed to protect wildlife and preserve the natural habitat. Amy Cooper (no relationship), a White woman falsely portrayed him as a threat after he asked her to comply with park rules by placing her dog on a leash. Irritated by the request, she retorted “I’m calling the cops…I’m gonna tell them there’s an African American man threatening my life.” Her deliberate emphasis on Christian Cooper’s race was intentional. It invoked a long, troubling and often deadly history in the United States for Black men who have been stereotyped as inherently dangerous, violent, and criminal, particularly to White women [32, 33]. The incident, recorded by Christian Cooper and widely circulated online, exposed the dangerous and the ever present intersection of race, fear, and power in America [34]. More broadly, it demonstrated how White identity and the social authority historically attached to it can be used to endanger Black people through appeals to law enforcement and racialized narratives of danger. Amy Cooper understood the cultural and historical weight attached to the image of a Black man allegedly threatening a White woman. Her performance on the phone call was theatrical and calculated; her voice became increasingly distressed as she sought to portray herself as a frightened victim. She weaponized a racial script in an attempt to gain power and protection through the police.

Christian Cooper’s experience illustrated how ordinary interactions can quickly become dangerous and the psychological burden many Black Americans carry in public spaces. Though the setting was a modern urban park and not a national park where Black visitors have historically been underrepresented, the incident nevertheless reflected the long-standing racial dynamics connected to White elite privilege and entitlement within parks, nature and open spaces [35]. Historically, access to pristine landscapes, recreational parks, and environmental amenities has often been shaped by race and class, with White Americans more readily viewed as legitimate occupants, stewards, and “protectors” of these spaces. The presence of Black Americans has often been met with suspicion, surveillance, or exclusion. As exemplified by the segregation of public swimming pools and beaches or national (e.g., Shenandoah National Park), regional and local parks during the Jim Crow era, public nature spaces have rarely functioned in a racially neutral capacity within the United States. Instead, public parks are some of the most “contentious and paradoxical places” that reflect broader systems of racial hierarchy and social control that have resulted in displacement, exploitation, and even death [36]. Other areas throughout the world influenced by the poison of colonization have also experienced such discrimination. For example, in South Africa, apartheid policies restricted access to parks, beaches, and other public recreational spaces based on racial classification, while the dispossession of Indigenous peoples in Australia and throughout the globe from their ancestral lands exterminated traditional cultural practices [37, 38]. The Central Park confrontation revealed how deeply embedded White supremacist notions of space persist in current day society. Amy Cooper’s response to Christian Cooper suggested an implicit belief that a Black man questioning her behavior in a shared natural environment represented an intrusion into a space where she felt socially entitled. She demonstrated how Whiteness can operate as a form of environmental privilege that grants some individuals the social power to define who belongs in parks and who appears suspicious or “out of place.” This entitlement is historically connected to the way outdoor recreation, conservation movements, and environmental spaces in America have long centered White experiences while marginalizing Black participation. Even the imagery associated with hiking, birdwatching, camping, and national park tourism has traditionally been framed through a White elitist lens, contributing to the perception that Black engagement in these activities are anomalies. Christian Cooper’s identity as a Black birdwatcher challenged the dominant racial norm and narrative of birdwatchers. According to 2011 U.S. Fish and Wildlife Service data, 4% of birdwatchers were Black American, but a more recent statistic from 2021 found that 0.5% of birdwatchers were Black American [39]. His mere presence in the Ramble not only disrupted unspoken racialized assumptions about ownership and belonging within natural and open spaces, but it also dared to impose another realization of his recreational identity. It highlighted how Black Americans can be hypervisible in predominantly White recreational settings, where ordinary actions such as walking, jogging, or “Birding While Black” may trigger heightened scrutiny. Ultimately, Christian Cooper’s experience exposed how the legacy of racial exclusion continues to shape who is presumed to belong in nature and who must constantly negotiate their legitimacy within it.

## “Walking While Black”: Access and Belonging

As mentioned previously, Black visitors in national parks have historically been underrepresented. Data from the National Park Service’s latest Socioeconomic Monitoring Visitor Survey reported that among visitors who completed surveys at 24 randomly selected parks in 2023, only 1% identified as Black American and collectively only 9% reported being from a minoritized race [40]. Shaped by slavery, segregation, racial violence, environmental inequality, and exclusionary cultural narratives, these data have been consistent over time [41]. As such, the concepts of nature access and nature belongingness are deeply intertwined with race, geography, public policy, and social power in the United States. While Black Americans have historically cultivated profound relationships with land, agriculture, forests, waterways, and outdoor recreation, systemic racism has often limited both physical access to natural spaces and the psychological sense of safety and inclusion within them [42, 43]. Therefore, understanding Black American nature access and belongingness requires examining the historical roots of exclusion, the environmental inequalities embedded in urban and rural landscapes, and the contemporary movements for reclaiming Black presence in nature.

While enslaved, the land for Black Americans was simultaneously a site of labor exploitation, punishment, survival, and resistance. They worked agricultural fields under brutal conditions while also using forests, rivers, and swamps as spaces of refuge and escape. “Walking While Black” inhabited a unique meaning when maroon communities and freedom seekers connected to the Underground Railroad relied on their intimate knowledge of natural landscapes to survive and evade capture [44, 45]. Following emancipation, racial terror and segregation further contoured Black experiences in outdoor spaces, particularly since newly freed men and women were now a “class of landless serfs” [46]. Sharecropping, an agricultural labor system that perpetuated racialized and economic hardship, upsurged after the Civil War and during the Reconstruction era. Many formerly enslaved Black Americans lacked land, money, and economic resources, while White southern landowners needed labor to rebuild their agricultural economy. Sharecropping was one of the dominant labor systems across the South in the late 19th century and well into the 20th century. During this period of perpetual servitude when 75% of Black Americans were sharecroppers, these farmers still found nature as a source of deep admiration [47, 48]. The rise and existence of the Jim Crow era, from the end of Reconstruction to the passage of major civil rights legislation in the 1960 s, also excluded Black Americans from many public parks, beaches, swimming pools, campgrounds, national parks and other outdoor spaces through formal laws and informal intimidation [36, 49–51]. In 1952, among 180 state parks catering to White Americans in nine southern states, only 12 of the parks allowed admission to Black Americans [52]. However, this was not only a southern practice. Hannibal Gerald Duncan, a PhD student at the University of Pennsylvania observed and documented this level of discrimination up North for his doctoral thesis [53].

“*Negroes are excluded from all of the popular parks in Cincinnati…in Chicago the White people, with ropes and guns, rid Gage Park of Negroes…at Clemington, New Jersey, the Atlantic City for the poor, Negroes are allowed to go there but one day in the year…in Boston the Negroes are practically excluded from many of the parks, playgrounds…according to law they can use them, but they feel better when they do not try to use them.”*[53]

Even when legalized segregation ended, unequal access persisted. Many Black neighborhoods were intentionally separated from green infrastructure and recreational amenities that were more common in affluent White suburbs. Separation was often instigated and craftily engineered by practitioners, like Robert Moses. One of the most influential urban planners of the 20th century, Moses used his position and influence to restrict Black Americans from using a wide range of public parks and recreational amenities, like Jones Beach, located on the south shore of Long Island, New York. For the “common people,” he believed that “they were lousy, dirty people, throwing bottles all over Jones Beach” and “for Negroes” in particular, “he considered [them] inherently dirty,” [54]. With tactics such as deliberately keeping the water in one swimming pool cold, employing only White lifeguards and attendants, or using bridge obstruction and mandated permits to discourage public transportation to parks, he barred poor, Black, Puerto Rican, and other non-White New Yorkers from accessing the city’s and region’s natural environments [54]. Furthermore, federal, state and local housing policies contributed to the concentration of Black communities in urban areas with fewer parks and less tree canopy [55]. Persistent disinvestment in nature or open spaces within Black communities was and still is ubiquitous throughout the country [56–59].

*“Robert Moses spent millions of dollars enlarging Riverside Park through landfill, but he did not spend a dime for that purpose between 125th and 155th streets. He added 132 acres to the parts of the park most likely to be used by White people—but not one acre to the part of the park most likely to be used by Black people.”*[54]

Today, research consistently shows that predominantly Black communities have less access to parks, trails, trees and natural amenities in terms of acreage and quality compared to predominantly White communities [55, 60]. Yet, the issue is not merely whether parks exist, but whether Black Americans feel welcomed and protected within those parks. Again, Black park users may experience surveillance, racial profiling, or suspicion while engaging in ordinary activities such as jogging, birdwatching, hiking, or resting in public spaces, which may in turn impact their desire to immerse themselves in the outdoors. Nature access refers not only to the presence of parks or green space, but also to the quality, safety, affordability, and usability of those spaces. Relatedly, nature belongingness refers to the feeling that one is accepted, safe, represented, and culturally connected within outdoor and environmental spaces. Mainstream American environmentalism has historically defined “wilderness” as a White elite construct that centered White experiences and aesthetics and marginalized Black environmental narratives [35]. National park histories, conservation imagery, and up until very recently outdoor recreation advertisements have frequently portrayed nature as a White domain. This exclusionary portrayal contributes to what Carolyn Finney describes as the “invisibility” of Black people in environmental discourse which not only whitewashes that narrative, but it effectively invokes a historical and contemporary erasure of Black Americans from the “Great Outdoors” [61]. As a result, many Black Americans have become alienated from outdoor cultures and even internalized nature as socially coded White spaces. The disruption of nature belongingness has been reinforced by historical trauma and intergenerational memory. Embedded in a collective remembrance are stories of racial violence in many natural settings. Until these spaces can be redefined and celebrated for Black Americans as peaceful, restorative, and therapeutic, nature belongingness for this group will continue to be a challenge.

## “Walking While Black”: Health and Well-being

**Fig. 4:**
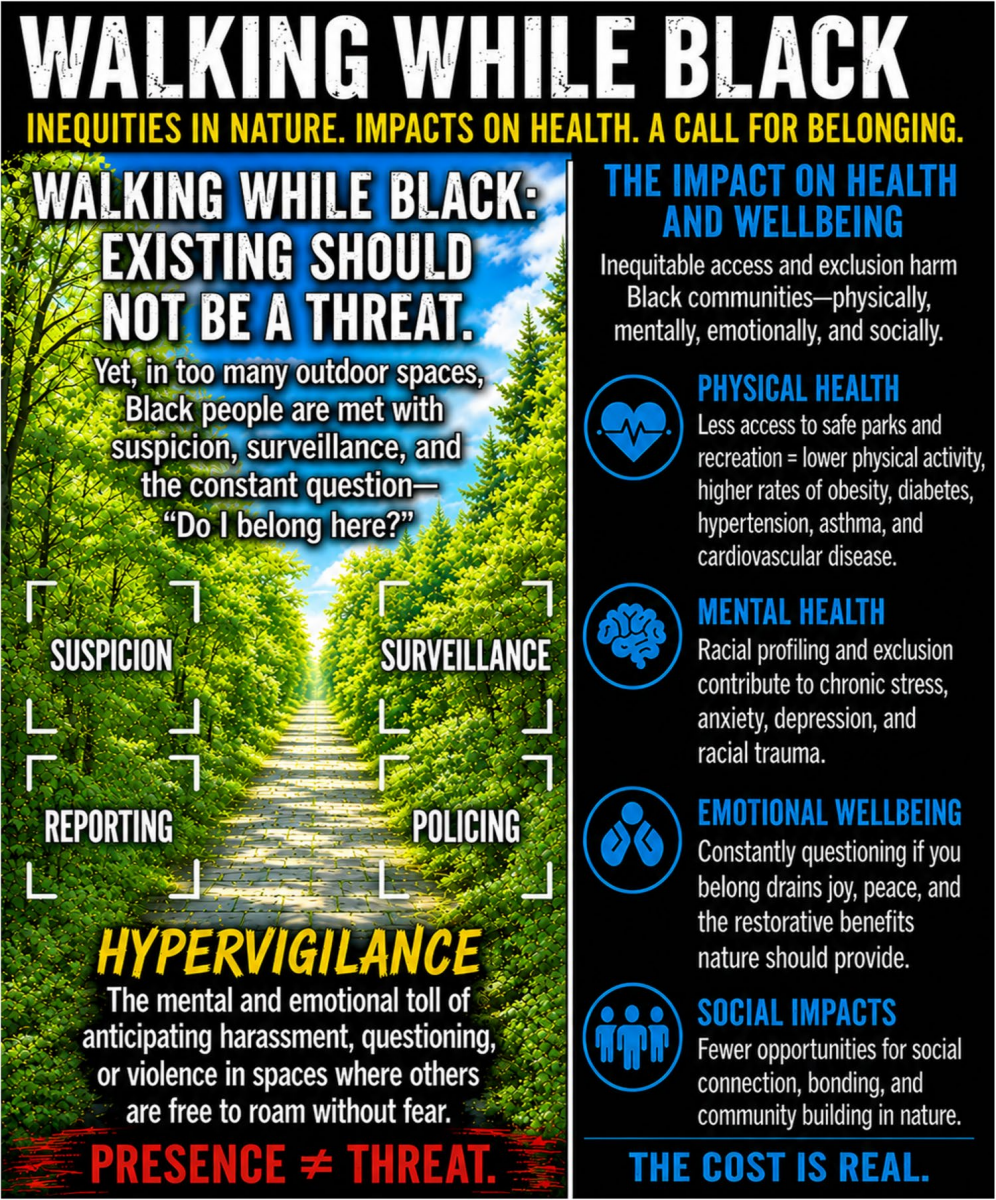
“Walking While Black”: Health and Wellbeing Impacts

Nature is increasingly understood not simply as wilderness but as a public health resource and a cultural space that should be accessible to all communities. Yet, unequal access to and sense of belongingness within nature and open spaces has profound implications for the health and wellbeing of Black Americans. The contemporary concept of “Walking While Black” captures this reality by describing the suspicion, surveillance, and potential danger Black people may encounter while simply existing in public spaces. Together, inequities in nature access and belongingness contribute to significant physical, psychological, emotional, and social health disparities among Black Americans (Fig. 4).

### Physical Health Impacts

Nature exposure is widely recognized as an important driver or determinant of physical health. Access to nature and green space is strongly associated with improved physical health outcomes, including lower rates of obesity, hypertension, cardiovascular disease, diabetes, and premature mortality [62–64]. Regular exposure to parks, trails, and open spaces encourages physical activity such as walking, jogging, cycling, and other forms of recreation, while also reducing stress-related physiological responses that contribute to chronic illness [65]. The bidirectional benefits of nature and physical activity are mutually reinforcing, as exposure to natural environments encourages movement and exercise, while physical activity in turn enhances the mental, emotional, and physiological health benefits derived from spending time in nature [66–69]. Communities with fewer parks and recreational opportunities often have lower rates of physical activity and higher rates of obesity and chronic disease [70, 71]. Safe sidewalks, tree-lined streets, trails, and recreational amenities encourage walking and exercise, but neighborhoods lacking such infrastructure often discourage active living. Within the context of “Walking While Black,” these physical health benefits are often undermined for Black Americans by racial profiling, surveillance, harassment, and fear of violence in public spaces. For some individuals, the simple act of walking through a park, jogging in a neighborhood, birdwatching, or exercising outdoors can carry the psychological burden of being perceived as suspicious or threatening. This chronic hypervigilance, a trauma driven state in which the nervous system stays on constant alert for danger, and stress response can elevate cortisol levels, increase blood pressure, contribute to inflammation, and discourage outdoor physical activity altogether [72–74]. Consequently, inequities in nature access and belongingness not only restrict opportunities for active living and recreation but also reinforce broader racial health disparities by limiting the physical health benefits that nature and open space are intended to provide. Structural inequities in nature access also intersect with broader environmental justice concerns. For example, predominantly Black communities are more likely to experience environmental hazards such as air pollution, urban heat islands, flooding, and industrial contamination while simultaneously lacking protective green infrastructure [75, 76]. Additionally, tree canopy inequities contribute to higher urban temperatures in historically redlined Black neighborhoods, increasing heat-related illness and mortality risks [77, 78]. One study found that Black residents (3.12 ± 2.67 °C) had the highest average enhancement in summer daytime surface temperature due to urban heat island intensity compared to Hispanic (2.70 ± 2.64 °C) and White residents (1.47 ± 2.60 °C) across 175 major U.S. cities” [78].

### Mental Health Impacts

Spending time in nature or open space significantly improves mental health by lowering stress hormones, reducing anxiety, and boosting mood [68, 79]. It has been found that just 120 min per week can improve cognitive function, increase feelings of happiness, and foster a sense of calm [80]. Key mechanistic theories, including the Biophilia Hypothesis, Stress Reduction Theory, and Attention Restoration Theory, have demonstrated how natural environments can diminish cognitive fatigue and activate the parasympathetic nervous system [81, 82]. However, when the relationship with nature for many Black Americans has been shaped by fear, hypervisibility, and social exclusion, this psychological burden can diminish the restorative benefits typically associated with nature exposure. High-profile incidents, such as the Christian Cooper incident mentioned previously, have highlighted the dangers associated with Black mobility and presence in natural environments. The 2020 killing of Ahmaud Arbery, another high-profile incident, underscored the life-threatening risks associated with “Running While Black” through public spaces. Consequently, the psychological consequences of these experiences are substantial for the individuals, families, and the Black community as a whole. Chronic exposure to racial profiling and environmental exclusion contributes to heightened stress, anxiety, hypervigilance, and racial trauma. Black Americans navigating predominantly White outdoor spaces may experience anticipatory stress, the constant awareness that they may be questioned, surveilled, or challenged regarding their presence. Nature has been found to improve connection and awe and foster a sense of being [83, 84]. Yet, the boosts of serotonin and dopamine can be undermined if there are perceptions of discrimination or social exclusion. Research demonstrates that perceived discrimination is associated with increased allostatic load, elevated cortisol levels, poor sleep quality, depression, and adverse cardiovascular outcomes [85, 86]. Thus, environments that should promote relaxation and restoration may instead become sites of fear and emotional exhaustion.

### Social Health Impacts

Nature and access to green space provide profound social health benefits by fostering social connection, strengthening communities, encouraging cultural expression, and creating opportunities for collective healing and belonging [68, 87, 88]. Parks, trails, playgrounds, community gardens, and other outdoor spaces often function as important social environments where families gather, neighbors interact, children play, elders share knowledge, and communities build trust and social cohesion. These interactions contribute to lower levels of social isolation, stronger support networks, improved communication among community members, and an increased sense of collective efficacy and safety. Nature-based spaces also provide opportunities for intergenerational bonding, cultural traditions, and spiritual reflection, all of which are associated with improved quality of life and stronger social wellbeing [89–91]. Research has shown that communities with accessible and welcoming green spaces often experience greater civic engagement, social capital, and emotional resilience during periods of stress and hardship [92, 93]. Outdoor recreation often serves as a mechanism for social cohesion, family bonding, community engagement, and identity formation [94, 95]. When Black Americans feel excluded from hiking, camping, birdwatching, or park visitation, because at one point in time they were legally prohibited from entering such spaces, opportunities for intergenerational connection to nature may be lessened. Opportunities for friendship formation, neighborhood interaction, mentorship, and family engagement in nature may also be reduced. As such, the social health benefits of nature and public outdoor spaces have been constrained by the long historical and contemporary realities associated with “Walking While Black,” “Birding While Black,” “Hiking While Black” or just “Existing While Black.” Inequities in access to safe, welcoming, and culturally inclusive natural spaces do not only impact physical and mental health but also undermine the social dimensions of health by limiting opportunities for community building, leisure, recreation, and social connectedness.

## Conclusion

Nature should not be a privilege reserved for some Americans while denied or contested for others. Inequities in nature access and belongingness are deeply intertwined within the broader history of racial inequality in the United States. The concept of “Walking While Black” reveals that mobility, recreation, and even presence within open spaces remain racialized experiences for many Black Americans. The health impacts of these inequities are multidimensional, encompassing physical illness, psychological distress, chronic stress, social exclusion, and diminished quality of life. Nature possesses enormous potential to support human health and wellbeing, yet its benefits cannot be fully actualized while racialized barriers to access, safety, and belonging persist. Achieving environmental and health equity therefore requires not only expanding access to green spaces but also transforming public spaces into environments where Black Americans can move, recreate, and exist freely without fear, suspicion, or exclusion. Community-based organizations and movements, such as Outdoor Afro and Black People Who Hike, have increasingly sought to reclaim and reconnect Black people with nature. They promote outdoor engagement, environmental education, and collective experiences of safety and empowerment in nature. These efforts challenge the dominant narrative regarding who belongs outdoors and they help to foster cultural affirmation and healing. Ultimately, ensuring equitable nature access and belonging is not only a public health issue, but a matter of racial justice.

## Key References

These eight references collectively provide the manuscript’s strongest foundation across four major domains: (1) historical origins of racialized surveillance, (2) inequities in nature access and belongingness, (3) environmental justice and park disparities, and (4) the physical and mental health consequences of exclusion from nature.

- Finney C. *Black Faces, White Spaces: Reimagining the Relationship of African Americans to the Great Outdoors.* University of North Carolina Press. 2014.
  - ○ This landmark book examines the historical and contemporary exclusion of Black Americans from environmental narratives and outdoor recreation. Finney’s work provides the foundational framework for understanding nature belongingness and the social construction of outdoor spaces as predominantly White environments.
- National Park Service (NPS). *2023 Socioeconomic Research of National Park Service Visitors: Report on 2023 Data Collection.* U.S. Department of the Interior. 2023.
  - ○ This report documents the persistent underrepresentation of Black Americans in U.S. national parks, providing contemporary evidence of racial disparities in nature visitation and access. The findings support the manuscript’s discussion of inequities in outdoor recreation participation.
- Center for American Progress (CAP). *The Nature Gap: Confronting Racial and Economic Disparities in the Destruction and Protection of Nature in America.* 2020.
  - ○ A seminal report demonstrating that communities of color have disproportionately less access to nature and green space. The report highlights structural inequities in environmental amenities and serves as a key reference for understanding environmental justice dimensions of nature access.
- Winkler RL, Clark JAG, Locke DH, et al. *Unequal Access to Social, Environmental and Health Amenities in U.S. Urban Parks.* Nature Cities. 2024;1(12):861–870.
  - ○ This contemporary study provides empirical evidence that access to urban park resources and associated health-promoting amenities remains unevenly distributed across racial and socioeconomic groups, reinforcing the manuscript’s arguments regarding environmental inequity.
- Hadden SE. *Slave Patrols: Law and Violence in Virginia and the Carolinas.* Harvard University Press. 2003.
  - ○ A foundational historical analysis of slave patrols and racialized surveillance. This work helps establish the manuscript’s historical argument that contemporary experiences of “Walking While Black” are rooted in longstanding systems of monitoring and controlling Black mobility.
- Roberts JD, Omidvar Tehrani S, Bratman GN. *Black Bodies and Green Spaces: Remembering the Eminence of Nature During a Pandemic.* In: *Sport and Physical Culture in Global Pandemic Times: COVID Assemblages.*Springer; 2023.
  - ○ This chapter explores the intersection of race, nature, and wellbeing, emphasizing the importance of outdoor environments for Black communities while highlighting persistent barriers to equitable access and belongingness in natural spaces.
- Jimenez MP, DeVille NV, Elliott EG, et al. *Associations Between Nature Exposure and Health: A Review of the Evidence.* International Journal of Environmental Research and Public Health. 2021;18(9).
  - ○ A comprehensive review summarizing the physical, mental, and social health benefits of nature exposure. This paper provides the scientific basis for understanding why inequitable access to nature contributes to health disparities.
- Smith NA, Voisin DR, Yang JP, Tung EL. *Keeping Your Guard Up: Hypervigilance Among Urban Residents Affected by Community and Police Violence.* Health Affairs. 2019;38(10):1662–1669.
  - ○ This study offers important evidence regarding hypervigilance and chronic stress associated with racialized surveillance and violence, helping explain the psychological and physiological consequences of “Walking While Black.”

## Data Availability

No datasets were generated or analysed during the current study.

## Footnotes

1 Throughout this review, Black American refers to descendants of enslaved Africans whose collective history has been shaped by successive systems of White supremacy and White-dominant social, political, and cultural institutions in the United States. This definition is intended to identify a specific population that is the focus of this analysis and should not be interpreted as excluding Black people throughout the diaspora who immigrated to the United States and who also experience racism and discrimination in this country.

2 Johnnie Cochran (1937–2005) was a renowned American trial attorney and civil rights advocate whose courtroom skills made him one of the most prominent legal figures in the nation. He achieved international recognition as the lead attorney of the defense team in the 1995 O.J. Simpson murder trial. Cochran also became known for his memorable courtroom argument, encapsulated in the phrase, “If it doesn’t fit, you must acquit.”

3 The Confederacy, formally known as the Confederate States of America, was a breakaway government formed by 11 southern states that seceded from the United States between 1860 and 1861. The Confederacy existed during the American Civil War and was created primarily to preserve and expand the institution of slavery.

4 Founded in 2023, Return to the Land (RTTL) is a controversial private membership association that operates a Whites-only, self-declared separatist community on a 160-acre settlement in rural Ravenden, Arkansas. The goal of RTTL is to create decentralized communities centered on traditional family structures, agrarian living, and what it calls "European heritage." The controversy surrounding RTTL stems from its explicit emphasis on racial ancestry. Critics, including civil rights advocates, contend that the project amounts to racial segregation and may conflict with the spirit or letter of fair housing laws.
